# Commentary: Somatic Stem Cells and Their Dysfunction in Endometriosis

**DOI:** 10.3389/fsurg.2017.00037

**Published:** 2017-07-14

**Authors:** Ekaterini Christina Tampaki, Athanasios Tampakis, Konstantinos Kontzoglou, Gregory Kouraklis

**Affiliations:** ^1^Second Department of Propaedeutic Surgery, Laiko General Hospital, School of Medicine, National and Kapodistrian University of Athens, Athens, Greece; ^2^Department of Visceral Surgery, Basel University Hospital, Basel, Switzerland

**Keywords:** somatic stem cells, endometriosis, mesenchymal to epithelial transition, epithelial to mesenchymal transition, stem cell niche

It was a pleasure to go through the manuscript by Dusan Djokovic and Carlos Calhaz-Jorge on somatic stem cells (SSCs) and their dysfunction in the pathogenesis of endometriosis (1). As EnSCs are adult stem cells comprising of an epithelial stem cells population, mesenchymal stem cells, and side population stem cells, they are of particular interest being implicated in angiogenesis and vascularization processes during tissue regeneration, being a steady supply of autologous stem cells for women and currently being tested in several clinical trials regarding their regenerative and therapeutic potential (2).

However, although we agree concerning the clarity of the data presented by the authors, we believe that there is always an association worth mentioning between the role of SSCs regarding the pathophysiology of endometriosis and the epithelial to mesenchymal transition (EMT) and mesenchymal to epithelial transition (MET) mechanisms when accumulating data are reviewed and presented regarding neoplastic and chronic inflammatory conditions such as endometriosis. A possible crucial connection between EMT and the stem cell niche, in other words the microenvironment favoring the disease, possibly all being involved in the pathogenesis of endometriosis is also a matter of great future investigation worth mentioning.

Indeed, a possible role of EnSCs related to the development of ovarian endometriosis and ovarian endometrioid carcinoma has been supported by a variety of studies as also suggested by Mirantes et al. (3). As already known, the endometrium normally deriving from the intermediate mesoderm, *via* MET taking place during the development of the urogenital system, could be prone to return to its original state *via* EMT, meaning endometrial epithelial cells returning to their mesenchymal origin and consequently being involved in the pathogenesis of pelvic endometriosis (4).

Epithelial to mesenchymal transition and MET-like processes are involved in the pathogenesis of endometriosis, as shed menstrual effluent can induce the EMT in mesothelial cells leading to phosphorylation cascades activation regarding proteins involved in cytoskeletal restructuring including a filament system transit from cytokeratin to vimentin, intermediate filaments, and microtubule system remodeling, affecting mesothelial cell shape and motility, energy metabolism through ATP, and metabolic activity. Furthermore, the connection between menstrual factor signals and the cytoskeletal reorganization is regulated by proteins activated during EMT affecting signal transduction and gene transcription (5). Moreover, the SP cells, exhibiting high efflux ability, develop into mesenchymal cell lineages (1), an expression of EMT activity, whereas menstruation could induce EMT pathways in endometrial stem cells and their progeny to migrate, invade, and establish endometriotic lesions through MET at ectopic sites.

Moreover, concepts like EMT, stem cell niche, and endometriosis pathogenesis need to be further investigated. More specifically, Barragan et al. in a late study came close to a conclusion that human endometrial stromal fibroblast deriving from normal resident endometrial mesenchymal stem cells promote an inflammatory phenotype as they develop progesterone resistance, that is a step toward characterization of the endometrial stem cell niche, a key element that will play in the future a major role in better understanding the microenvironment favoring the disease and will lead us to an in-depth characterization of the enSCs and subsequently endometriosis (6).

Furthermore, Eggers et al. found in a late study that downregulation of microRNA miR-200b is observed in endometriosis and malignant disease, driving tumor cells toward an invasive state by enhancing EMT. Interestingly, miR-200b upregulation may inhibit EMT and invasive growth in endometriosis, consequently affecting proliferation, invasiveness, and stemness of endometriotic cells by targeting ZEB1, ZEB2, and KLF4. The authors also demonstrated that cell proliferation and the stemness-associated side population phenotype were enhanced following miR-200b transfection. These properties were possibly attributed to the upregulation of the pluripotency-associated transcription factor KLF4 and require attention when considering therapeutic strategies. In conclusion, upregulation of miR-200b reverses EMT, emerging as a potential therapeutic approach to inhibit endometriotic cell motility and invasiveness (7).

Besides the progenitor stem cells residing in the uterus, mesenchymal stem cells may also travel from the bone marrow to repopulate the progenitor population as principle source of endometriosis outside of the peritoneal cavity differentiating into endometriosis in ectopic locations (1). These cells can differentiate into epithelial cells, consequently demonstrating that cells of extra uterine origin also regulate the ectopic endometrium and furthermore indicating the potential of stem cells into regenerating or repairing the tissue after endometrial injury or a chronic inflammation such as endometriosis (8). Interestingly, this is another factor underlying the crucial role and connection between the EMT and MET mechanisms along the SSCs going hand in hand when examining their function in endometriosis, as ectopic stem cell trafficking could possibly result in pathology (8). Indeed, the re-supply of endometrium with bone marrow-derived stem cells could be the key in understanding the cellular basis for the widely non-beneficial und unsuccessful use of conservative alternatives to hysterectomy (9). Interestingly, in murine models where male-to-female bone marrow transplants were performed, it has been demonstrated that the ability of male bone marrow not being able to supply circulating endometrial cells and produce *de novo* endometrial cells, stems from mesenchymal stem cells, meaning that bone marrow-derived mesenchymal stem cells are traditionally being involved in repair after injury and possibly ectopic implantation (8, 10).

The traditional theory for the origin of endometriosis in ectopic locations, also suggested by Djocovic et al., is based on retrograde menstruation from the fallopian tubes with ectopic implantation. However, this theory does not fully explain endometriosis occurrence in areas away from the peritoneal cavity, where retrograde menstruation and mechanisms of lymphovascular dissemination, direct transplantation, abnormal migration, and invasion cannot justify this tissue presence. On the other hand, the existence of a circulating source of ectopic stem cell engraftment meaning bone marrow-derived cells differentiating into endometrial tissue in ectopic locations could possibly suggest a novel mechanism for the etiology of endometriosis (8). Therefore, we believe that there is always an association worth mentioning between the role of SSCs regarding the pathophysiology of endometriosis and the EMT and MET mechanisms as in a variety of far removed areas presenting endometriosis, to bone marrow-derived stem cell homing a differentiation EMT and MET mechanisms are also implicated (8, 10), especially when accumulating data are reviewed and presented regarding such conditions.

## Author Contributions

ECT conceived of the idea and wrote the manuscript. AT helped draft the manuscript. KK helped to revise the manuscript. GK helped to revise the manuscript. All authors read and approved the final manuscript.

## Conflict of Interest Statement

The authors declare that the research was conducted in the absence of any commercial or financial relationships that could be construed as a potential conflict of interest.

## Abbreviations

SSCs, somatic stem cells; EnSCs, endometrial stem cells; EMT, epithelial to mesenchymal transition; MET, mesenchymal to epithelial transition; eSF, endometrial stromal fibroblasts; eMSCs, endometrial mesenchymal stem cells; EPCs, endothelial progenitor cells.
